# Synovial fluid proteomic biomarkers in periprosthetic joint infection: A systematic review with gene ontology and protein network analyses

**DOI:** 10.1002/jeo2.70853

**Published:** 2026-07-20

**Authors:** Giorgia Lucia Benedetto, Elvira Immacolata Parrotta, Raffaele Covello, Giovanni Cuda, Giorgio Gasparini, Olimpio Galasso, Umile Giuseppe Longo, Michele Mercurio

**Affiliations:** ^1^ Department of Medical and Surgical Sciences University “Magna Graecia” Catanzaro Italy; ^2^ Department of Experimental and Clinical Medicine University “Magna Graecia” Catanzaro Italy; ^3^ Department of Orthopaedic and Trauma Surgery “Magna Græcia” University, “Renato Dulbecco” University Hospital Catanzaro Italy; ^4^ Research Center on Musculoskeletal Health, MusculoSkeletalHealth@UMG Magna Graecia University Catanzaro Italy; ^5^ Department of Medicine, Surgery and Dentistry University of Salerno Salerno Italy; ^6^ Fondazione Policlinico Universitario Campus Bio‐Medico Roma Italy; ^7^ Research Unit of Orthopaedic and Trauma Surgery, Department of Medicine and Surgery Università Campus Bio‐Medico di Roma Rome Italy

**Keywords:** biomarkers, cytokines, inflammation, MS/MS, PJI, PPI network, proteomics, synovial fluid

## Abstract

**Purpose:**

Periprosthetic joint infection (PJI) is a severe complication of total joint arthroplasty associated with significant morbidity, implant failure and increased healthcare costs. Diagnosis remains challenging, particularly in low‐grade and culture‐negative infections, because conventional markers lack specificity. Proteomic analyses may identify more reliable synovial biomarkers and provide insights into the molecular mechanisms underlying PJI.

**Methods:**

A systematic review was conducted according to PRISMA guidelines using PubMed and Scopus databases. Studies published within the last 10 years involving adults undergoing revision hip or knee arthroplasty for suspected PJI and assessing synovial fluid biomarkers by proteomic or immunoassay techniques (liquid chromatography–tandem mass spectrometry, matrix‐assisted laser desorption/ionisation time‐of‐flight mass spectrometry, enzyme‐linked immunosorbent assay) were included. Functional enrichment analyses (gene ontology [GO], Kyoto encyclopaedia of genes and genomes [KEGG], human phenotype ontology [HPO], DISEASES and protein–protein interaction (PPI) network analyses were performed.

**Results:**

Six studies met the inclusion criteria. A focused set of dysregulated synovial proteins was identified, predominantly related to innate immunity and inflammation, including lactoferrin, myeloperoxidase, lysozyme C, PRTN3, defensin alpha 1, defensin alpha 3, calprotectin (S100 calcium‐binding proteins A8 and A9), annexin A6, alpha‐2‐HS‐glycoprotein (Fetuin‐A), MNDA, GRO‐α, interleukin (IL)‐8 and IL‐5. GO and KEGG analyses demonstrated enrichment of antimicrobial defense and cytokine‐mediated inflammatory pathways. PPI analysis identified key hub proteins involved in inflammatory and oxidative stress responses, while HPO and DISEASES analyses further supported their association with immune dysregulation and infectious inflammatory conditions.

**Conclusions:**

Synovial proteomic and immuno‐inflammatory biomarkers may improve diagnostic accuracy in PJI, particularly in low‐grade and culture‐negative infections. Biomarker panels including α‐defensins and calprotectin (S100A8/A9) represent promising next‐generation diagnostic tools. Further multicenter studies are needed to validate these findings and facilitate their integration into standardised diagnostic algorithms.

**Level of Evidence:**

Levels II–III.

**Abbreviations**
AHSG: alpha‐2‐HS‐glycoprotein (Fetuin‐A)
ANXA6: annexin A6
CXCL: C‐X‐C motif chemokine ligand
DEFA1/3: defensin alpha 1/3 (Human Neutrophil Peptides 1–3, HNP1–3)
ECM: extracellular matrix
ELISA: enzyme‐linked immunosorbent assay
FDR: false discovery rate
GO: gene ontology
HPO: human phenotype ontology
IL: interleukin
IL‐5: interleukin 5
IL‐8(CXCL8): interleukin 8 (C‐X‐C motif chemokine ligand 8)
KEGG: Kyoto encyclopaedia of genes and genomes
LC‐MS/MS: liquid chromatography–tandem mass spectrometry
LTF: lactoferrin
LYZ: lysozyme C
MPO: myeloperoxidase
MSIS: musculoskeletal Infection Society
PJI: periprosthetic joint infection
PPI: protein–protein interaction
RETN: resistin
S100A8/A9: calprotectin (S100 calcium‐binding proteins A8 and A9)
STRING: search tool for the retrieval of interacting genes/proteins

## INTRODUCTION

Periprosthetic joint infection (PJI) is one of the most severe complications following joint arthroplasty and remains a leading cause of implant failure, revision surgery and morbidity worldwide [25]. The incidence of PJI after primary arthroplasty is estimated at 1%–2% for hip and knee replacements, with even higher rates reported following revision procedures [12, 15]. Diagnosis remains challenging due to the heterogeneous clinical presentation and the limitations of conventional culture‐based methods [28]. Currently, laboratory criteria rely on traditional serological and synovial markers, including C‐reactive protein (CRP), erythrocyte sedimentation rate (ESR), synovial leucocyte count and polymorphonuclear percentage [17, 27]. However, these parameters lack specificity, may be confounded by non‐infectious inflammatory conditions, and often fail to detect low‐grade infections or cases involving prior antibiotic exposure [11, 16]. This diagnostic uncertainty underscores the need for novel biomarkers with improved sensitivity and specificity. Recent proteomic and immunoassay‐based studies have identified several promising candidate biomarkers across synovial fluid, sonicate fluid, blood and periprosthetic tissue. Among the most consistently reported are antimicrobial peptides and neutrophil‐derived proteins such as α‐defensins (DEFA1, DEFA3; also known as HNP1–3), lactoferrin (LTF), myeloperoxidase (MPO) and lysozyme C (LYZ), reflecting the central role of neutrophil activation and innate immunity in PJI pathogenesis [10, 31]. Additional mediators include S100 proteins (S100A8 and S100A9), which act as alarmins to amplify local inflammation, and annexin A6 (ANXA6), involved in membrane repair and vesicle trafficking [33]. Furthermore, cytokines and chemokines such as GRO‐α, interleukin (IL)‐8 and IL‐5, have been associated with PJI, highlighting robust pro‐inflammatory signalling and immune cell recruitment [7]. Metabolic and systemic biomarkers, including resistin (RETN) and alpha‐2‐HS‐glycoprotein (AHSG), have also been implicated, suggesting links between systemic inflammation, host metabolism and implant‐associated infection [30]. Collectively, these findings underscore the complex interplay among innate immunity, adaptive responses and extracellular matrix remodelling in the pathophysiology of PJI. Despite rapid advances in biomarker discovery, a systematic synthesis of proteomic biomarkers for PJI remains lacking. This review aims to summarise current evidence on proteomic and inflammatory biomarkers with diagnostic and prognostic potential in PJI and evaluate their translational applicability in clinical practice.

## MATERIALS AND METHOD

### Search strategy

A systematic literature search was conducted and reported in accordance with the Preferred Reporting Items for Systematic Reviews and Meta‐Analyses (PRISMA) guidelines [9]. PubMed and Scopus databases were searched for studies published within the past 10 years (2015–2025). The search was restricted to the last 10 years to capture the most recent and methodologically comparable studies, reflecting advances in proteomic and immunoassay technologies. The search aimed to identify studies evaluating synovial fluid biomarkers for the diagnosis of PJI following total hip arthroplasty (THA) or total knee arthroplasty (TKA). Search terms combined keywords and Boolean operators (AND/OR/NOT) were used to refine the search, including: ‘periprosthetic joint infection’, ‘PJI’, ‘synovial fluid’, ‘proteomics’, ‘mass spectrometry’, ‘LC‐MS’, ‘MALDI‐TOF’, ‘cytokine assay’, ‘ELISA’, ‘extracellular vesicles’ and ‘biomarker’.

### Inclusion criteria and study selection

The eligibility criteria for study inclusion were defined according to the PICO framework: [6] Population (P): adult patients (>18 years) with suspected PJI after THA or TKA; intervention/exposure (I): Measurement of synovial fluid biomarkers using proteomic or immunoassay‐based techniques (e.g., MS/MS, LC‐MS/MS, matrix‐assisted laser desorption/ionisation time‐of‐flight mass spectrometry [MALDI‐TOF], multiplex cytokine assays, ELISA, extracellular vesicle analysis); Comparator (C): regardless of the presence or absence of comparator or control groups, all studies were eligible; Outcome (O): Diagnostic performance of biomarkers for identifying PJI, identification of reproducible biomarkers distinguishing PJI from aseptic failure, and correlations with conventional inflammatory parameters. Only original research articles published in English and conducted in human populations were included. Exclusion criteria comprised animal or in vitro studies, investigations focused solely on genetic or epigenetic markers without corresponding proteomic or biomarker analyses, case reports, reviews, editorials, technical notes and studies enroling fewer than ten patients per group. However, excluded studies were screened for potential relevance during qualitative synthesis and discussion. The study selection process was conducted in two stages. In the first phase, three independent reviewers screened all titles and abstracts retrieved from the database search according to the predefined eligibility criteria. Potentially relevant articles were subsequently evaluated in full text to confirm inclusion. Discrepancies were resolved through discussion and when necessary, adjudicated by a fourth reviewer.

### Data extraction and quality assessment

Data extraction from the selected studies was performed independently by three reviewers (M.M., G.L.B. and E.I.P), who systematically collected bibliographic information and methodological details. For each study, the following data were recorded: first author, year of publication, study design, clinical characteristics of included patients and type of surgical procedure. Particular attention was given to the type of sample analysed, synovial fluid in all cases, and the analytical techniques employed, which included proteomic methods such as LC–MS/MS and MALDI–TOF, as well as multiplex cytokine assays, ELISA and extracellular vesicle analyses. Both proteomic and immunoassay‐based studies were included, as they represent complementary and widely used approaches for the assessment of synovial fluid protein biomarkers. Although based on different analytical principles, both methodologies were considered relevant to capture the current landscape of biomarker research in PJI. In addition to identifying the biomarkers under investigation, data were extracted on their diagnostic performance, including sensitivity, specificity, predictive values and area under the curve (AUC), along with correlations to conventional synovial parameters such as white blood cell (WBC) count, polymorphonuclear cell percentage and calprotectin levels. To ensure methodological rigour, all observational studies were appraised using the Newcastle–Ottawa Scale (NOS), which evaluates three domains: participant selection, group comparability and exposure/outcome assessment, with a maximum score of nine points. One randomised controlled trial (RCT) [19] was identified and assessed separately using the RoB 2 tool, which evaluates bias across five domains: randomisation, deviations from intended interventions, missing data, outcome measurement and selective reporting.

### Data analysis

Owing to the methodological heterogeneity among the included studies, particularly regarding sample size, analytical techniques and outcome measures, a qualitative synthesis of the findings was conducted. The identified biomarkers were grouped and compared on the basis of their biological function and the type of study. Biomarkers reported in multiple studies were further examined to identify recurring molecular patterns. Only significantly dysregulated proteins reported in the included studies were considered for downstream bioinformatic analyses, while non‐significant proteins were excluded. To explore their potential biological roles and associated pathways, a functional enrichment analysis was performed via ShinyGO v0.76 (http://bioinformatics.sdstate.edu/go/), an online bioinformatics platform. This tool enables the classification of biomarkers according to gene ontology (GO) categories, namely biological processes, molecular functions and cellular components and facilitates the mapping of these biomarkers to known molecular and disease‐related pathways through Kyoto encyclopaedia of genes and genomes (KEGG) analysis. Statistical significance was assessed via the false discovery rate (FDR), and only terms and pathways with an FDR‐adjusted *p*‐value below a defined threshold (FDR < 0.05) were considered significantly enriched. In addition to GO and KEGG analyses, human phenotype ontology (HPO) enrichment was performed using ShinyGO to identify potential associations between the identified biomarkers and known clinical phenotypes. This approach allowed the detection of links between specific biomarkers and phenotypic traits relevant to the disorders under investigation, offering a more comprehensive understanding of their possible clinical relevance. Furthermore, protein‐protein interaction networks were explored via the STRING database (version 12.0), which visualises functional relationships among the identified biomarkers and highlights potential clusters or hubs of molecular interaction. In addition, STRING was also used to perform Disease–gene association (DISEASES) enrichment analysis. ShinyGO was used for functional enrichment analysis to assess over‐represented GO terms and KEGG pathways. STRING was used to construct and analyse protein–protein interaction networks based on known and predicted interactions.

## RESULTS

### Characteristics of included studies

**Figure 1:**
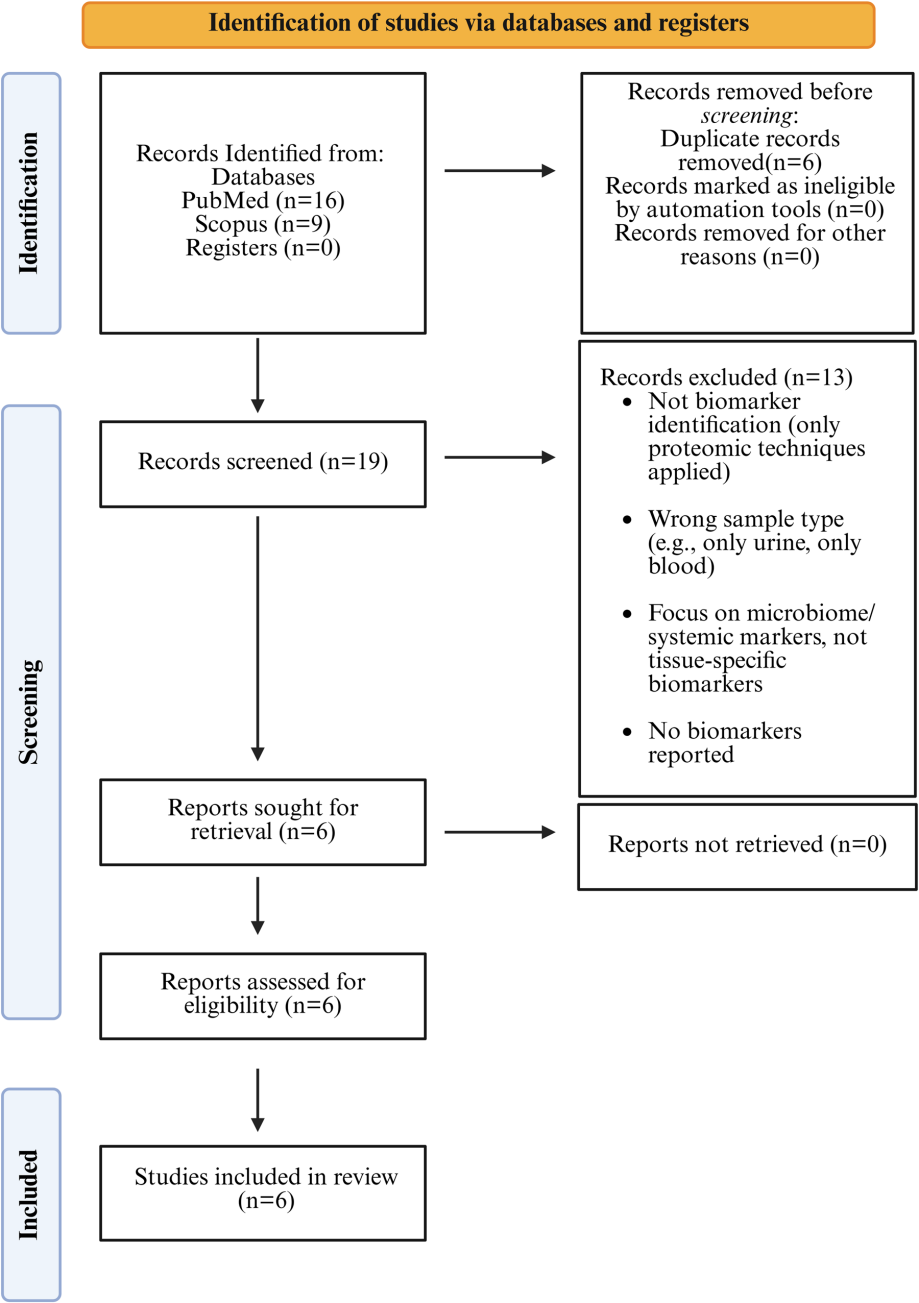
PRISMA 2020 flow diagram depicting the study selection process. The diagram shows the number of records identified through database searches (PubMed and Scopus), duplicates removed, records screened, full‐text articles assessed for eligibility and the final number of studies included in the review, based on predefined inclusion and exclusion criteria.

A total of six studies met the inclusion criteria and were included in this systematic review. The study selection process is presented in the PRISMA flow diagram (Figure 1). The selected studies encompass a range of methodological designs, including one RCT [24] and several non‐randomised clinical observational case‐control studies [2, 3, 8, 23, 29]. The methodological quality of the included studies was assessed using the Newcastle‐Ottawa Scale (NOS) for observational studies and the Cochrane Risk of Bias 2 (RoB 2) tool for the RCT. All six observational studies achieved a NOS score of 6/9, indicating moderate methodological quality. Common limitations included relatively small sample sizes, single‐centre study designs, and incomplete adjustment for potential confounding factors. The RCT [33] was evaluated using the RoB 2 tool and was judged to have an overall low RoB, with some concerns related to the reporting of the randomisation process. The detailed quality assessment is summarised in Table 1. Most investigations focused on the proteomic analysis of synovial fluid. The proteomic techniques applied across studies were highly heterogeneous. For downstream analyses, only significantly dysregulated proteins identified in the selected studies were included, while non‐significant proteins were excluded. MS/MS and LC‐MS/MS were used for the quantification of specific proteins such as S100A8, S100A9, α‐defensins, LTF and neutrophil‐derived proteins. Other approaches included multiplex cytokine analyses and MS/MS (MALDI‐TOF), often combined with ELISA for biomarker validation. Sample sizes ranged from 30 to 200 patients, reflecting different recruitment strategies and study designs. Key identified biomarkers include α‐defensins, S100A8/S100A9, IL‐8, LTF, PRTN3 and MNDA, while some studies highlighted biomarker patterns associated with specific pathogens. Overall, the findings suggest that combining multiple biomarkers, often alongside conventional inflammatory parameters, improves diagnostic accuracy for the management of PJI. An overview of the main characteristics of the included studies is provided in Table 2.

**Table 1:** Risk of bias and methodological quality assessment of included studies.

Newcastle‐Ottawa Scale (NOS)						
Study	Design	Selection (0–4)	Comparability (0–2)	Exposure (0–3)	Total Score (0–9)	
Sallai et al. [23]	Observational, case‐control	3	1	2	6	
Argyrou et al. [2]	Observational, case‐control	3	1	2	6	
Balato et al. [3]	Observational, prospective diagnostic	3	1	2	6	
Iorio et al. [8]	Observational, diagnostic, case–control	3	1	2	6	
Wang et al. [29]	Retrospective diagnostic	3	1	2	6	
**ROB 2.0**						
**Study**	**Randomisation process**	**Deviations from intended interventions**	**Missing data**	**Measurement of outcomes**	**Selection of reported results**	**Overall RoB2 judgement**
Xu et al. [33]	Some concerns	Low risk	Low risk	Low risk	Low risk	Some concerns

*Note*: Observational studies were assessed using the NOS, which evaluates Selection, Comparability, and Outcome/Exposure domains, with scores ranging from 0 to 9. Higher scores indicate better methodological quality. The randomised controlled trial was evaluated using the Cochrane Risk of Bias 2 (RoB 2) tool, and judgements are reported for each bias domain and overall risk of bias.

**Table 2:** Characteristics of included proteomic studies evaluating synovial fluid biomarkers for prosthetic joint infection (PJI).

Study	Study type	Patients (PJI/non‐PJI)	Sample type	Proteomic technique	Identified biomarkers	Key findings
Xu et al. [33]	RCT‐DB	39/43	SF	MS/MS	S100A8, S100A9, α‐Defensin	All markers were significantly elevated in PJI (*p* < 0.001).
Sallai et al. [23]	OBS‐CC	17/17	SF, EVs	MS/MS	Lactotransferrin, Myeloperoxidase, Lysozyme C, Annexin A6, alpha‐2‐HS‐glycoprotein (AHSG)	Significantly altered proteins between study groups: lactotransferrin (*p* = 0.00646), myeloperoxidase (*p* = 0.01061), lysozyme C (*p* = 0.04687), annexin A6 (*p* = 0.03921) and AHSG (*p* = 0.03146).
Argyrou et al. [2]	OBS‐CC	14(+1 likely infected)/15	SF	Multiplex cytokine (73‐plex assay panel)	growth‐regulated oncogene alpha (GROA), interleukin‐8, interleukin‐5, S100‐A8/calprotectin and resistin (RETN)	GRO‐α, IL‐8, IL‐5, S100‐A8/calprotectin and resistin showed significant diagnostic performance (all *p* < 0.05).
Balato et al. [3]	OBS‐DX	33/92	SF	LC‐MS/MS	α‐Defensin	High diagnostic accuracy for PJI: AUC 0.99; sensitivity 100%, specificity 97%
Iorio et al. [8]	OBS‐DX	59/79	SF	MALDI‐TOF MS	α‐Defensin	Accuracy 94.9%, sensitivity 93%, specificity 96%, AUC 0.95
Wang et al. [29]	RETRO	25/25	SF	ELISA, MS/MS	Lactoferrin (LTF), Polymorphonuclear leucocyte serine protease 3 (PRTN3), Myeloid nuclear differentiation antigen (MNDA)	LTF, PRTN3, MNDA promising biomarkers; high AUCs (0.9488–0.9888); ELISA confirmed sensitivity and specificity

*Note*: This table summarises the study design, sample characteristics, proteomic techniques used, identified synovial fluid biomarkers, types of comparison groups and key outcomes for PJI. Biomarkers were primarily measured in synovial fluid using MS/MS, LC‐MS/MS, MALDI‐TOF MS, ELISA, or multiplex cytokine analysis.

Abbreviations: ELISA, enzyme‐linked immunosorbent assay; EVs, extracellular vesicles; LC‐MS/MS, liquid chromatography‐tandem mass spectrometry; MALDI‐TOF MS, matrix‐assisted laser desorption/ionisation time‐of‐flight mass spectrometry; MS/MS, tandem mass spectrometry; Multiplex, Multiplex cytokine analysis; Obs‐CC, observational, case‐control; Obs‐Dx, observational, diagnostic accuracy study; RCT, randomised controlled trial; Retro, retrospective study; SF, synovial fluid.

### Identified biomarkers

Across the included studies, a wide range of proteomic biomarkers have been identified in synovial fluid, most of which are associated with inflammatory responses, innate immunity and neutrophil activation. Key proteins include S100A8, S100A9, α‐defensins (DEFA1, DEFA3 and HNP1‐3), LTF, MPO, LYZ and ANXA6, as well as various cytokines and chemokines such as IL‐8, IL‐5 and GRO‐α (CXCL1). A smaller subset of proteins, including AHSG, was consistently elevated in non‐infectious conditions, highlighting their potential as negative biomarkers. Most biomarkers were detected in synovial fluid, reflecting the local joint environment during PJI. Several proteins, including S100A8/calprotectin, DEFA1/3 and LTF, demonstrated strong correlations with conventional inflammatory parameters, such as %PMNs and neutrophil activation, and exhibited high diagnostic accuracy, with AUC values ranging from 0.90 to 0.99 for α‐defensins. Extracellular vesicle‐associated proteins, including MPO, LYZ and ANXA6, were also found to be significantly elevated in PJI, suggesting their role in the pathophysiology of infection. Table 3 provides an overview of the most commonly reported synovial fluid biomarkers, the direction of change in PJI versus non‐PJI, and relevant correlations.

**Table 3:** Common synovial fluid biomarkers identified across studies.

Biomarker	Identified in studies	Direction in PJI versus non‐PJI	Notes/correlations
S100A8	Xu et al.; Argyrou et al. [2, 32]	Increase in PJI	Correlates with %PMNs and calprotectin; high diagnostic performance
S100A9	Xu et al.; Argyrou et al. [2, 32]	Increase in PJI	Often co‐expressed with S100A8; part of calprotectin complex
DEFA1	Xu et al.; Balato et al.; Iorio et al.[3, 8, 32]	Increase in PJI	High diagnostic accuracy
LTF (Lactoferrin)	Sallai et al.; Wang et al. [23, 29]	Increase in PJI	Associated with PMN activation; high sensitivity and specificity
DEFA3	Xu et al. [32]	Increase in PJI	High diagnostic accuracy
MPO (Myeloperoxidase)	Sallai et al. [23]	Increase in PJI	Correlates with neutrophil activity; part of PMN‐derived EVs
LYZ (Lysozyme C)	Sallai et al. [23]	Increase in PJI	PMN‐related protein; part of innate immune response
ANXA6 (Annexin A6)	Sallai et al. [23]	Increase in PJI	Associated with extracellular vesicles in PJI
AHSG (Alpha‐2‐HS‐glycoprotein)	Sallai et al. [23]	Increase in non‐PJI	Negative acute phase protein; inversely correlates with CRP
PRTN3	Wang et al. [29]	Increasein PJI	Potential synovial fluid biomarkers for PJI diagnosis
MNDA	Wang et al. [29]	Increase in PJI	Potential synovial fluid biomarkers for PJI diagnosis
HNP 1‐3	Xu et al. [32]		Provide satisfactory diagnostic utility for PJI diagnosis
GRO‐α (CXCL1)	Argyrou et al. [2]	Increasein PJI	Chemokine involved in neutrophil recruitment; diagnostic relevance
CXCL8 (IL‐8)	Argyrou et al. [2]	Increase in PJI	Cytokine involved in inflammation regulation
IL‐5	Argyrou et al. [2]	Increase in PJI	Immune regulatory cytokine; moderate diagnostic value
RETN (Resistin)	Argyrou et al. [2]	Increase in PJI	Immune regulatory protein; moderate diagnostic value

*Note*: This table summarises the main synovial fluid biomarkers reported in studies on periprosthetic joint infection (PJI). For each biomarker, the corresponding studies, direction of change in PJI versus non‐PJI, and relevant diagnostic notes or correlations are indicated. Biomarkers were identified through proteomic analyses and related immunoassays.

Abbreviations: CRP, C‐reactive protein; EVs, extracellular vesicles; PMNs, polymorphonuclear neutrophils.

### Outcomes of functional enrichment analysis

**Figure 2:**
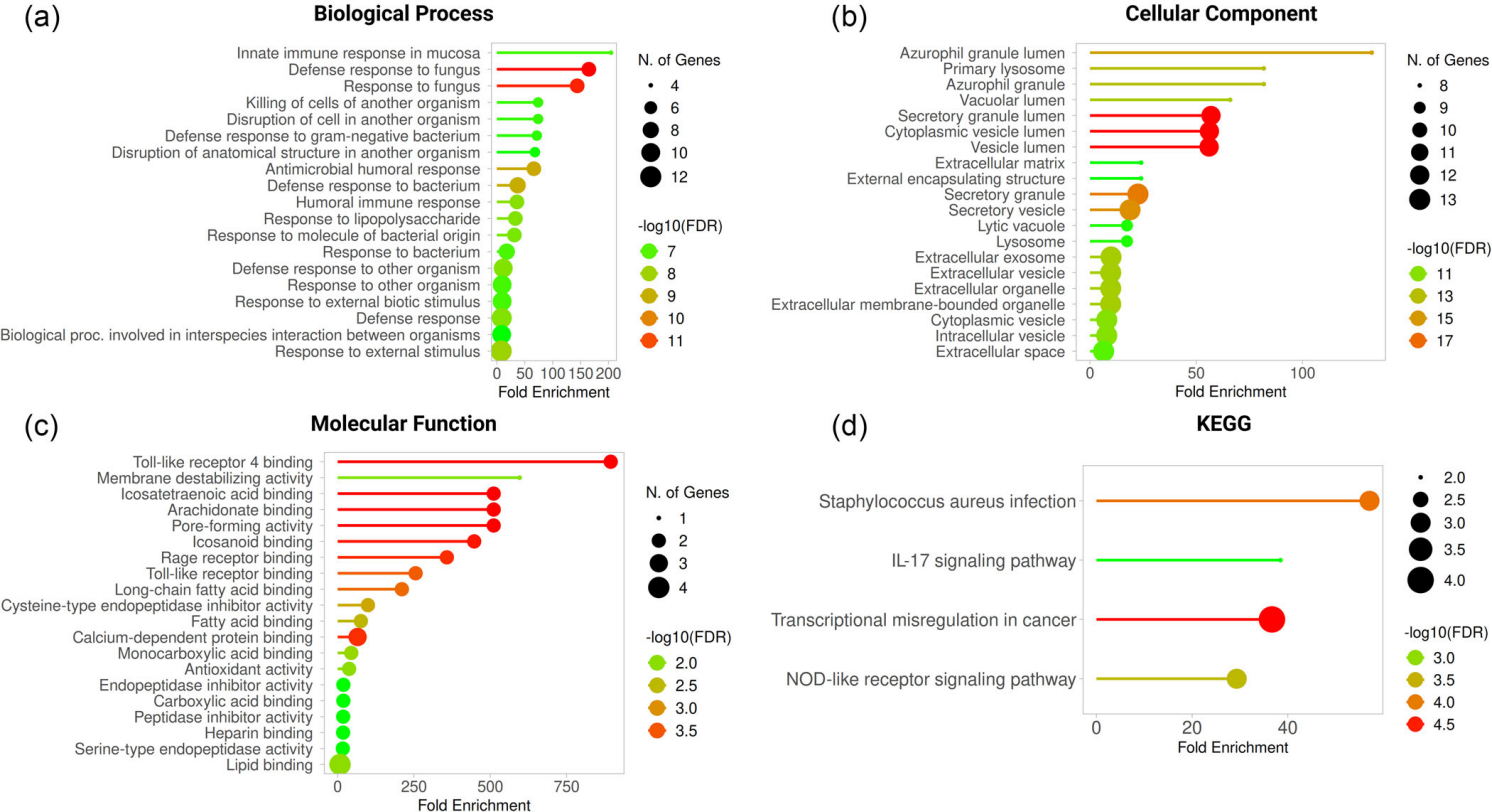
Functional enrichment analysis of the most frequently identified proteomic biomarkers in periprosthetic joint infection (PJI). (a–c) Gene ontology (GO) enrichment analysis of biological processes, cellular components and molecular functions. (D) Kyoto encyclopaedia of genes and genomes (KEGG) pathway enrichment analysis. Analyses were performed using ShinyGO v0.76 with false discovery rate (FDR)‐adjusted *p*‐values < 0.05.

**Figure 3:**
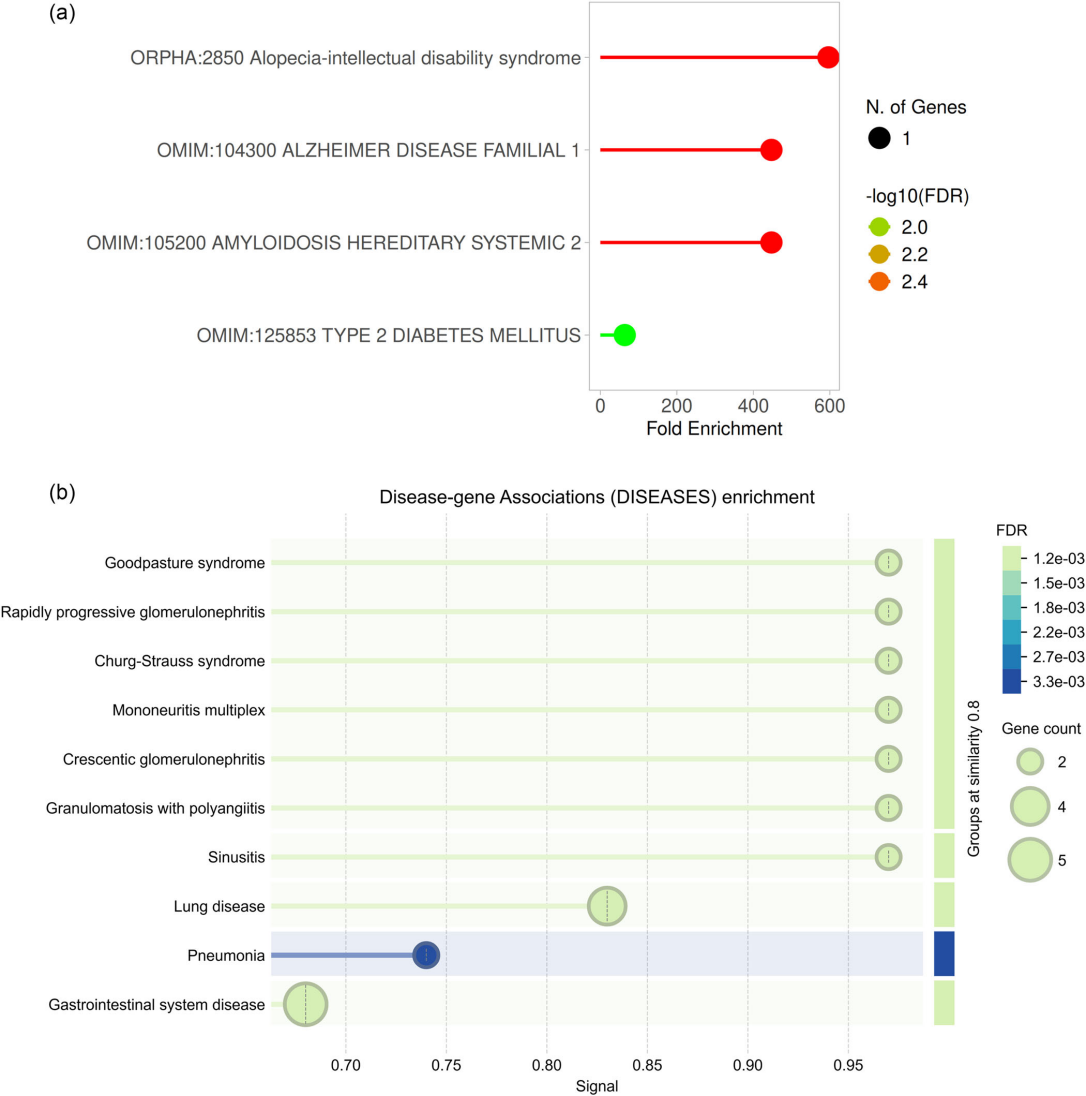
Phenotype and disease enrichment analyses of the identified proteomic biomarkers. (a) The figure shows the distribution of enriched disease phenotypes associated with the proteins. Analyses were performed using ShinyGO v0.76, with significance determined by false discovery rate (FDR)‐adjusted *p*‐values (FDR < 0.05). (b) DISEASES database enrichment analysis by STRING (combined score > 0.7).

Following the identification of candidate proteomic biomarkers, enrichment analyses were performed to explore their biological significance. GO analysis revealed strong enrichment in biological processes related to the immune and inflammatory response. Notably, terms such as *response to external stimulus, humoral immune response, innate immune response in mucosa, response to lipopolysaccharide and antimicrobial humoral response* (Figure 2a). For cellular components, enriched terms were primarily associated with the extracellular and secretory compartments, such as *secretory granule lumen*, *azurophil granule lumen, lysosome, extracellular matrix and secretory vesicle* (Figure 2b), consistent with the secreted and extracellular nature of many of the proteins implicated. In the molecular function category, the most significantly enriched terms included *toll‐like receptor 4 binding, calcium‐dependent protein binding, long‐chain fatty acid binding and antioxidant activity* highlighting the predominant involvement of immune mediators and signalling regulators (Figure 2c). Complementary KEGG pathway analysis identified significant associations with key signalling cascades relevant to inflammation and tissue remodelling, including the *IL‐17 signalling pathway, Staphylococcus aureus infection, transcriptional misregulation in cancer and NOD‐like receptor signalling pathway* (Figure 2d). These pathways converge on mechanisms of immune activation, matrix degradation and tissue repair, all central to the pathogenesis of PJI [13, 20]. The complete GO, KEGG and enrichment output datasets generated through ShinyGO are provided in File S3. In addition, HPO enrichment analysis (Figure 3a) linked the identified biomarkers to a spectrum of disease phenotypes. Associations were observed with conditions characterised by immune dysregulation and chronic inflammation, including Alopecia‐intellectual disability syndrome, Amyloidosis hereditary systemic 2, Alzheimer's disease familial 1 and Type 2 Diabetes Mellitus. Detailed HPO enrichment results are available in File S4. To further assess the clinical relevance of the identified proteins, disease enrichment analysis was performed using the DISEASES database (by STRING). Significant associations were observed with several inflammatory and immune‐mediated disorders, including granulomatosis with polyangiitis, crescentic glomerulonephritis, rapidly progressive glomerulonephritis, eosinophilic granulomatosis with polyangiitis (Churg–Strauss syndrome), mononeuritis multiplex, pneumonia, sinusitis, lung disease and gastrointestinal system disease. These associations were primarily driven by proteins involved in neutrophil degranulation, antimicrobial defense and inflammatory signalling, particularly MPO, PRTN3, LTF, S100A8, S100A9 and CXCL8 (Figure 3b). Detailed results of the disease enrichment analysis are reported in File S5. These analyses were performed to identify shared biological processes, pathways and disease associations across studies, providing an integrative interpretation beyond narrative synthesis.

### Protein–protein interaction (PPI) network analysis

**Figure 4:**
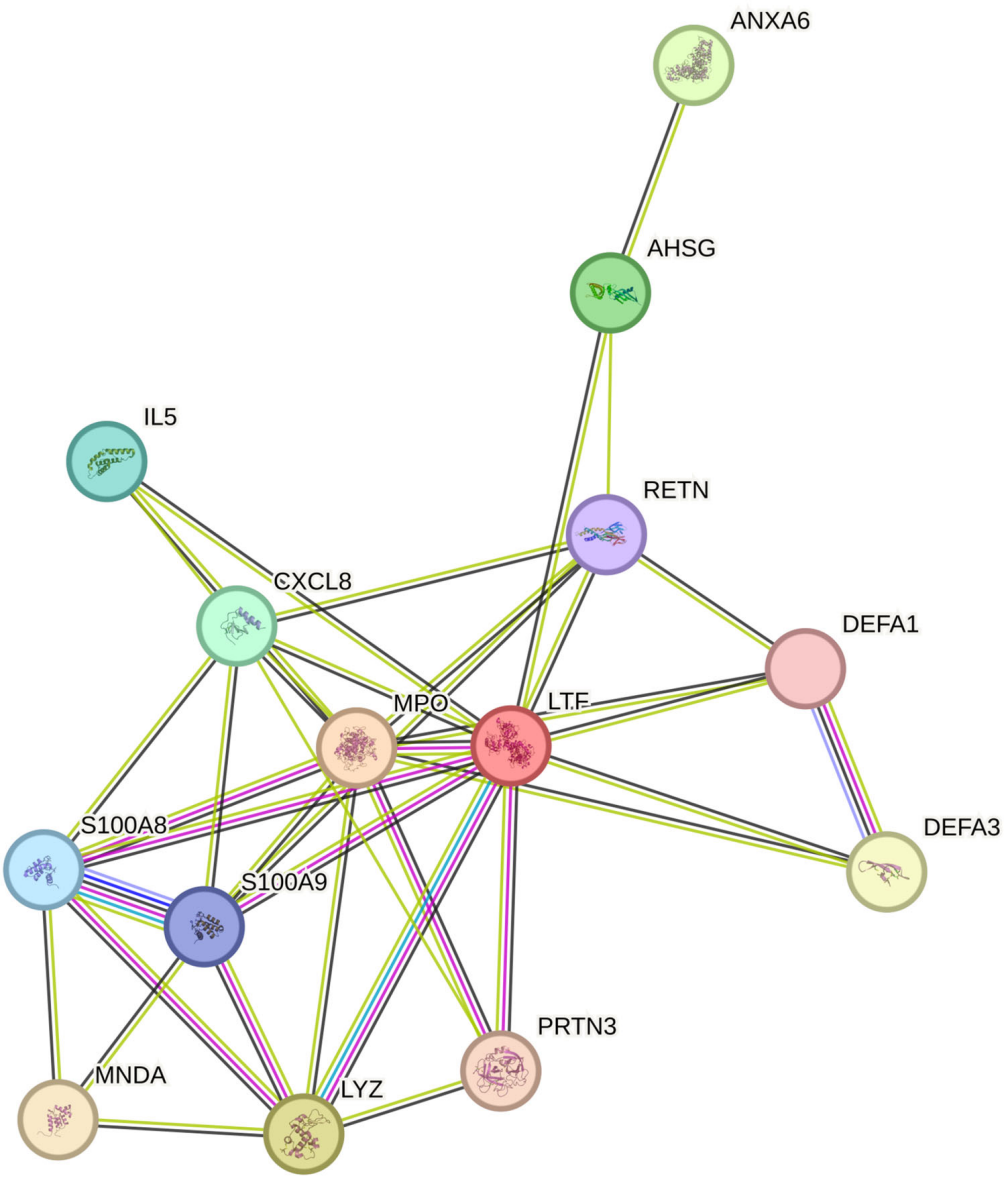
Protein–protein interaction (PPI) network of the identified periprosthetic joint infection (PJI)‐associated biomarkers generated using STRING v12.0. Nodes represent proteins and edges represent high‐confidence interactions (combined score > 0.7). Node size reflects the degree of connectivity within the network.

To further elucidate the molecular interplay among the proteomic biomarkers identified in patients with PJI, PPI and disease enrichment analyses were performed. The PPI network analysis generated using the STRING database (version 12.0). The PPI network included the proteins consistently identified across the selected studies (LTF, MPO, LYZ, ANXA6, AHSG, CXCL8, IL5, S100A8, S100A9, RETN, DEFA1, PRTN3, MNDA and DEFA3) and was filtered using a high‐confidence interaction score (>0.7). The resulting network (Figure 4) revealed a highly interconnected cluster centred on LTF, MPO, CXCL8, S100A8 and S100A9, which emerged as the principal hub proteins. These molecules displayed extensive interactions with antimicrobial effectors (DEFA1, DEFA3, PRTN3 and LYZ) and inflammatory mediators, highlighting their coordinated role in neutrophil activation, antimicrobial defense and cytokine‐mediated immune responses. In contrast, AHSG and ANXA6 occupied more peripheral positions within the network, suggesting participation in more specialised regulatory processes. An overview of the biological functions and molecular characteristics of the identified biomarkers, based on UniProt and GeneCards annotations, is provided in Table 4. The complete STRING interaction network output and associated interaction data are provided in File S5. Collectively, these results illustrate a complex network of immune and inflammatory mediators that cooperate to sustain the pathophysiological cascade of PJI.

**Table 4:** Functional overview of key proteins (Uniprot and GeneCards).

Gene	Protein	Primary Function
LTF	Lactotransferrin	Multifunctional iron‐binding glycoprotein with antimicrobial, immunomodulatory and bone‐regulatory activities
MPO	Myeloperoxidase	Neutrophil‐derived antimicrobial enzyme involved in reactive oxygen species–mediated microbial killing and vascular protection
LYZ	Lysozyme C	Bacteriolytic enzyme involved in innate immune defense and monocyte–macrophage function
ANXA6	Annexin A6	Protein involved in CD21 association and intracellular calcium regulation
AHSG	Alpha‐2‐HS‐glycoprotein	Calcium‐binding protein involved in endocytosis, opsonization and regulation of bone mineralisation
GRO‐α (CXCL1)	Growth‐regulated alpha protein	Chemokine with neutrophil chemotactic activity involved in inflammation and endothelial cell signalling; processed forms show increased activity
IL‐8 (CXCL8)	Interleukin‐8	Chemokine involved in inflammatory response, mediating neutrophil, basophil and T‐cell chemotaxis and neutrophil activation via CXCR1/CXCR2 signalling
IL‐5	Interleukin 5	T‐cell and NK cell–derived cytokine involved in eosinophil survival, differentiation and chemotaxis, and in B‐cell activation and immunoglobulin production
S100A8	S100 Calcium Binding Protein A8	Pro‐inflammatory calcium‐ and zinc‐binding alarmin involved in leucocyte recruitment, innate immune activation and antimicrobial activity
S100A9	S100 Calcium Binding Protein A9	Calcium‐ and zinc‐binding alarmin involved in regulation of inflammation, innate immune activation, leucocyte chemotaxis and antimicrobial defense
RETN	Resistin	Hormone linking metabolic dysfunction with immune cell chemotaxis and insulin resistance
DEFA1 (HNP‐1)	Defensin α−1	Innate immune effector with broad antimicrobial activity (bacteria and viruses), antiviral effects and immunomodulatory functions including activation of innate immune responses
PRTN3	Proteinase 3	Serine protease mediating extracellular matrix degradation and neutrophil migration/inflammatory responses
MNDA	Myeloid cell nuclear differentiation antigen	Transcriptional regulator in myeloid lineage involved in interferon‐mediated immune responses
DEFA3 (HNP‐3)	Defensin α−3	Antimicrobial effector of the innate immune system active against bacteria and viruses

*Note*: Functional overview of key proteins identified in the protein–protein interaction (PPI) network. This table summarises the main biological roles of the most relevant proteins identified through proteomic studies and included in the STRING‐based PPI network analysis. The listed proteins are involved in diverse but interconnected biological processes, including innate immune defense, inflammation, cytokine signalling, oxidative stress response and tissue homoeostasis. Functional annotations were derived from UniProt and GeneCards databases.

## DISCUSSION

This systematic review synthesises current evidence on proteomic biomarkers identified in synovial fluid from patients with PJI. The most consistently identified biomarkers include neutrophil‐derived antimicrobial peptides and enzymes such as α‐defensins (DEFA1 and DEFA3), LTF, MPO and LYZ, alongside members of the S100 protein family (S100A8 and S100A9). GO and KEGG pathway analysis highlighted enrichment in pathways involved in inflammation and tissue remodelling, including humoral immune response, calcium‐dependent protein, antioxidant activity, IL‐17 signalling pathway and NOD‐like receptor signalling. PPI network analyses showed the molecular complexity of PJI, revealing CXCL8, S100A8, S100A9 and MPO as major hub proteins forming a densely interconnected core of immune mediators and antimicrobial proteins. In the current systematic review, proteins most commonly reported represent the molecular hallmark of neutrophil activation and innate immune defense, a defining characteristic of PJI [1, 14]. The concurrent upregulation of proinflammatory cytokines and chemokines, including IL‐5, IL‐8 (CXCL8) and GRO‐α, further underscores the intense immune activation that occurs within the periprosthetic microenvironment. This cytokine‐chemokine axis orchestrates leucocyte recruitment, bacterial clearance and tissue remodelling, forming a self‐perpetuating inflammatory circuit [22]. Notably, IL‐8, GRO‐α and IL‐5 are key cytokines associated with the acute‐phase response and synovial neutrophilia, suggesting their potential role as dynamic indicators of infection severity [21, 26]. Functional enrichment analyses (GO and KEGG) corroborate these findings, revealing significant enrichment in biological processes linked to immune activation, antimicrobial response, oxidative stress and cytokine‐mediated signalling. KEGG pathways such as *Staphylococcus aureus infection and IL‐17 signalling*, reflecting the convergence of immune defense and inflammatory tissue injury in PJI [8, 9, 10, 11, 12, 13, 14, 15]. Taken together, these observations align with prior transcriptomic and metabolomic studies highlighting neutrophil‐driven inflammation and oxidative burst as central mechanisms of implant‐associated infection. In parallel, HPO and DISEASES analysis provided complementary insights, linking the identified protein set to phenotypic features associated with autoimmune and inflammatory disorders such as type 2 diabetes mellitus, Alzheimer's disease and hereditary amyloidosis [25]. Moreover, the presence of markers like AHSG (fetuin‐A), with known roles in metabolic regulation and anti‐inflammatory activity, points to possible compensatory mechanisms modulating excessive inflammation in the periprosthetic niche [23]. PPI network analyses further illustrated the molecular complexity of PJI, revealing IL‐5, CXCL8, S100A8, S100A9 and MPO as major hub proteins forming a densely interconnected core. These hubs represent functionally integrated pathways bridging innate immunity, oxidative stress and cytokine signalling. The central hub nodes formed a tightly connected module linking innate immune response, coordinating neutrophil activation and shaping the inflammatory microenvironment of PJI. Peripheral nodes such as AHSG and ANXA6 may exert modulatory effects, balancing immune activation and tissue protection. From a translational perspective, the identification of highly connected hub proteins may provide mechanistic insight into potential diagnostic or therapeutic targets, particularly those occupying nodal positions that could exert upstream regulatory control. This systems‐level perspective underscores the relevance of PPI network analysis in contextualising proteomic findings and in advancing the molecular understanding of PJI pathogenesis. By mapping proteomic biomarkers onto interaction networks, it may be possible to delineate hierarchical signalling relationships and to highlight candidate intervention points with potential translational relevance for future biomarker discovery or targeted therapeutic strategies.

Building on these systems‐level observations, the integration of proteomic data with transcriptomic and metabolomic analyses could further improve biological resolution and may enable the development of multi‐omics diagnostic frameworks that better capture the complexity of the inflammatory cascade in PJI and help identify early‐stage candidate biomarkers. Such integrative approaches might also contribute to a better understanding of inter‐individual variability in susceptibility. Moreover, these findings may support the future development of multi‐marker diagnostic panels combining neutrophil activation markers (e.g., α‐defensins, calprotectin) with cytokines (IL‐5 and IL‐8) to potentially improve diagnostic sensitivity and specificity, particularly in culture‐negative infections or infections caused by low‐virulence pathogens [4, 5, 15]. In this context, integrating proteomic markers with established diagnostic criteria, such as the Musculoskeletal Infection Society (MSIS) score or ICM definitions, could potentially refine diagnostic performance and support earlier detection strategies [29]. Additionally, proteomic profiles might, in future applications, help distinguish acute from chronic PJI or contribute to treatment stratification by identifying molecular signatures associated with persistent biofilm activity or systemic inflammation [18, 20].

### Strengths and limitations

This systematic review provides a comprehensive synthesis of proteomic biomarkers identified in synovial fluid from patients with PJI, integrating evidence across multiple studies and highlighting consistently reported immune‐related proteins, including neutrophil‐derived antimicrobial peptides, S100 proteins and key inflammatory mediators. A major strength of this study is the integration of functional enrichment analyses (GO and KEGG), PPI network mapping and phenotype‐based analysis (HPO), which together provide a systems‐level understanding of the molecular mechanisms underlying PJI. This multi‐layered analytical approach allowed the identification of coordinated biological processes involving innate immune activation, cytokine signalling and inflammatory network organisation. Furthermore, the identification of central hub proteins within the PPI network, such as IL‐5, CXCL8, S100A8, S100A9 and MPO, supports the robustness of the findings and highlights potential biologically relevant targets for future translational applications. Importantly, this study represents one of the first systematic reviews in this field to combine proteomic evidence with network‐based functional interpretation. Several limitations should be acknowledged. The number of available proteomic studies on PJI remains limited, and most included studies are observational in design with relatively small sample sizes. Considerable heterogeneity exists in study populations, sample processing methods and proteomic platforms, which may contribute to variability in protein identification and quantification across studies. In addition, differences in analytical workflows and reporting standards limit the direct comparability of results and may introduce methodological bias. Another is the methodological heterogeneity introduced by the inclusion of both proteomic and immunoassay‐based studies, which may differ in sensitivity, dynamic range and target specificity. The specificity of several identified inflammatory biomarkers is also limited, as many proteins, including cytokines such as IL‐5 and chemokines such as CXCL8 and, are not specific to PJI and may be elevated in other inflammatory or autoimmune conditions. Furthermore, the lack of standardised proteomic protocols and the absence of large, multicenter validation cohorts limit the generalisability and clinical translation of the findings. Finally, although network and enrichment analyses provide valuable systems‐level insights, they are based on existing database annotations and do not replace experimental validation.

## CONCLUSION

This systematic review synthesises current evidence on proteomic biomarkers identified in synovial fluid from patients with PJI. The most consistently identified biomarkers include neutrophil‐derived antimicrobial peptides and enzymes such as α‐defensins (DEFA1 and DEFA3), LTF, MPO and LYZ. Functional analysis highlighted enrichment in pathways involved in inflammation and tissue remodeling, including IL‐17 signalling, chemokine‐cytokine interaction. Protein network analyses showed the molecular complexity of PJI, revealing IL‐5, CXCL8, S100A8, S100A9 and MPO as major hub proteins forming a densely interconnected core of immune mediators and antimicrobial proteins. While current results remain exploratory, they provide a strong rationale for further biomarker validation, large‐scale multicenter studies and harmonisation of proteomic methodologies. Ultimately, proteomics‐driven precision diagnostics hold the potential to transform early detection, risk stratification and personalised treatment of PJIs, improving patient outcomes and healthcare efficiency.

## AUTHOR CONTRIBUTIONS

Elvira Immacolata Parrotta, Giorgia Lucia Benedetto and Michele Mercurio contributed equally to the conception, and writing of the manuscript. Giovanni Cuda, Giorgio Gasparini and Umile Giuseppe Longo provided critical revisions and conceptual guidance. Umile Giuseppe Longo, Raffaele Covello, Giorgia Lucia Benedetto and Michele Mercurio contributed to the data analysis and interpretation. Olimpio Galasso, Giorgio Gasparini, Michele Mercurio and Elvira Immacolata Parrotta supervised the project and provided final approval of the manuscript. All authors read and approved the final version of the manuscript.

## CONFLICT OF INTEREST STATEMENT

The authors declare no conflicts of interest.

## ETHICS STATEMENT

This article is a systematic review of previously published studies. Ethical approval and participant consent were obtained from the original studies, as stated by the respective authors. No new data involving human participants were collected or analysed by the authors of this review.

## Supporting information

Supplementary File 1: screening study.

Supplementary File 2: Input.

Supplementary File 3: enrichment GO and KEGG analysis results (ShinyGO output.xlsx format).

Supplementary File 4: enrichment HPO analysis (ShinyGO output.xlsx format).

Supplementary File 5: enrichment DISEASES analysis from STRING (tsv. format).

Supplementary File 6: Protein‐protein interaction network table exported from STRING (tsv. format).

## Data Availability

All data generated or analysed during this study are included in this published article and its supplementary information files. The supplementary information files include: File S1, study screening process; File S2, input dataset used for enrichment analyses; File S3, gene ontology (GO) and Kyoto encyclopaedia of genes and genomes (KEGG) enrichment results (ShinyGO output,.xlsx format); File S4, human phenotype ontology (HPO) enrichment results (ShinyGO output,.xlsx format); File S5, DISEASES enrichment analysis generated using STRING (.tsv format) and File S6, protein–protein interaction (PPI) network tables exported from STRING (.tsv format).

## References

[1] Akcaalan S , Ozaslan HI , Caglar C , Şimşek ME , Citak M , Akkaya M . Role of biomarkers in periprosthetic joint infections. Diagnostics. 2022;12:2958. 36552965 10.3390/diagnostics12122958 PMC9777153

[2] Argyrou C , Papagrigorakis E , Tzefronis D , Pliaka V , Fotis C , Kamariotis S , et al. Multiplex cytokine analysis for the identification of novel potential synovial fluid biomarkers for periprosthetic joint infections. Injury. 2024;55:111659. 38917741 10.1016/j.injury.2024.111659

[3] Balato G , Dall'anese R , Balboni F , Ascione T , Pezzati P , Bartolini G , et al. Synovial fluid alpha‐defensin in periprosthetic knee infection workup: liquid chromatography‐mass spectrometry detection of alpha‐defensin in synovial fluid. Bone Jt J. 2022;104–B:1047–1051. 10.1302/0301-620X.104B9.BJJ-2021-1672.R1 36047027

[4] Bizzoca D , Moretti L , Gnoni A , Moretti FL , Scacco S , Banfi G , et al. The usefulness of synovial fluid proteome analysis in orthopaedics: focus on osteoarthritis and periprosthetic joint infections. J Funct Morphol Kinesiol. 2022;7:97. 36412759 10.3390/jfmk7040097 PMC9680387

[5] Bonanzinga T , Ferrari MC , Tanzi P , Vandenbulcke F , Zahar A , Marcacci M . The role of alpha defensin in prosthetic joint infection (PJI) diagnosis: a literature review. EFORT Open Rev. 2019;4:10–13. 30800475 10.1302/2058-5241.4.180029 PMC6362339

[6] Bourebaba L , Marycz K . Pathophysiological implication of Fetuin‐A glycoprotein in the development of metabolic disorders: a concise review. J Clin Med. 2019;8:2033. 31766373 10.3390/jcm8122033 PMC6947209

[7] Erdemli B , Anıl Özbek E , Başarir K , Ceren Karahan Z , Öcal D , Biriken D . Proinflammatory biomarkers' level and functional genetic polymorphisms in periprosthetic joint infection. Acta Orthop Traumatol Turc. 2018;52:143–147. 29305046 10.1016/j.aott.2017.11.002 PMC6136306

[8] Iorio R , Viglietta E , Mazza D , Petrucca A , Borro M , Iolanda S , et al. Accuracy and cost‐effectivenss of a novel method for alpha defensins measurement in the diagnosis of periprosthetic joint infections. J Arthroplasty. 2021;36:3275–3281. 34088569 10.1016/j.arth.2021.05.013

[9] Keemu H , Vaura F , Maksimow A , Maksimow M , Jokela A , Hollmén M , et al. Novel biomarkers for diagnosing periprosthetic joint infection from synovial fluid and serum. JB JS Open Access. 2021;6:e20.00067. 10.2106/JBJS.OA.20.00067 PMC8154383 34056503

[10] Kelly MP , Darrith B , Hannon CP , Nam D , Courtney PM , Della Valle CJ . Synovial fluid alpha‐defensin is an adjunctive tool in the equivocal diagnosis of periprosthetic joint infection. J Arthroplasty. 2018;33:3537–3540. 30017218 10.1016/j.arth.2018.06.026

[11] Kunutsor SK , Whitehouse MR , Lenguerrand E , Blom AW , Beswick AD ; INFORM Team . Re‐infection outcomes following one‐ and two‐stage surgical revision of infected knee prosthesis: a systematic review and meta‐analysis. PLoS One. 2016;11:e0151537. 26967645 10.1371/journal.pone.0151537 PMC4788419

[12] Kurtz SM , Lau EC , Son M‐S , Chang ET , Zimmerli W , Parvizi J . Are we winning or losing the battle with periprosthetic joint infection: trends in periprosthetic joint infection and mortality risk for the medicare population. J Arthroplasty. 2018;33:3238–3245. 29914821 10.1016/j.arth.2018.05.042

[13] Li Z , Li Z‐Y , Maimaiti Z , Yang F , Fu J , Hao L‐B , et al. Identification of immune infiltration and immune‐related biomarkers of periprosthetic joint infection. Heliyon. 2024;10:e26062. 38370241 10.1016/j.heliyon.2024.e26062 PMC10867348

[14] Mazzaracchio V , Vitiello R , Maccauro G , Arduini F . Point‐of‐care devices for the detection of biomarkers of periprosthetic joint infection: state of the art and future perspectives. Trends Anal Chem. 2024;172:117544.

[15] Mercurio M , Castioni D , Iannò B , Gasparini G , Galasso O . Outcomes of revision surgery after periprosthetic shoulder infection: a systematic review. J Shoulder Elbow Surg. 2019;28:1193–1203. 31003887 10.1016/j.jse.2019.02.014

[16] Mercurio M , Castioni D , Porco E , Familiari F , Gasparini G , Galasso O . Periprosthetic ankle infection: eradication rate, complications, and limb salvage. A systematic review. Foot Ankle Surg. 2022;28:550–556. 34321185 10.1016/j.fas.2021.07.009

[17] Mercurio M , Galasso O , Familiari F , Iannò B , Bruno CF , Castioni D , et al. Trend of perioperative CRP (C‐reactive protein) levels in non‐infected total knee arthroplasty. Orthop Rev. 2022;14:36589. 10.52965/001c.36589 PMC9242728 35782199

[18] Mercurio M , Gasparini G , Cofano E , Zappia A , Familiari F , Galasso O . Knee arthrodesis for periprosthetic knee infection: fusion rate, complications, and limb salvage‐a systematic review. Healthcare. 2024;12:804. 38610226 10.3390/healthcare12070804 PMC11011444

[19] Moher D , Liberati A , Tetzlaff J , Altman DG ; PRISMA Group . Preferred reporting items for systematic reviews and meta‐analyses: the PRISMA statement. PLoS Med. 2009;6:e1000097. 21603045 PMC3090117

[20] O'Connor K , Koscianski C , Larson N , Mangalaparthi KK , Hoffmann C , Bedard NA , et al. Proteomic profile at the time of surgery correlates with disease stage and surgical outcome in periprosthetic joint infection. mBio. 2025;16:e0170025. 40874775 10.1128/mbio.01700-25 PMC12505968

[21] Piuzzi NS , Klika AK , Lu Q , Higuera‐Rueda CA , Stappenbeck T , Visperas A . Periprosthetic joint infection and immunity: current understanding of host‐microbe interplay. J Orthop Res. 2024;42:7–20. 37874328 10.1002/jor.25723

[22] Prince N , Penatzer JA , Dietz MJ , Boyd JW . Localized cytokine responses to total knee arthroplasty and total knee revision complications. J Transl Med. 2020;18:330. 32867801 10.1186/s12967-020-02510-w PMC7461261

[23] Sallai I , Marton N , Szatmári A , Kittel Á , Nagy G , Buzás EI , et al. Activated polymorphonuclear derived extracellular vesicles are potential biomarkers of periprosthetic joint infection. PLoS One. 2022;17:e0268076. 35533148 10.1371/journal.pone.0268076 PMC9084519

[24] Schardt C , Adams MB , Owens T , Keitz S , Fontelo P . Utilization of the PICO framework to improve searching PubMed for clinical questions. BMC Med Inform Decis Mak. 2007;7:16. 17573961 10.1186/1472-6947-7-16 PMC1904193

[25] Schindler M , Walter N , Maderbacher G , Sigmund IK , Alt V , Rupp M . Novel diagnostic markers for periprosthetic joint infection: a systematic review. Front Cell Infect Microbiol. 2023;13:1210345. 37529352 10.3389/fcimb.2023.1210345 PMC10388554

[26] Shahi A , Parvizi J . The role of biomarkers in the diagnosis of periprosthetic joint infection. EFORT Open Rev. 2016;1:275–278. 28461959 10.1302/2058-5241.1.160019 PMC5367543

[27] Sigmund IK , Puchner SE , Windhager R . Serum inflammatory biomarkers in the diagnosis of periprosthetic joint infections. Biomedicines. 2021;9:1128. 34572314 10.3390/biomedicines9091128 PMC8467465

[28] Vrancianu CO , Serban B , Gheorghe‐Barbu I , Czobor Barbu I , Cristian RE , Chifiriuc MC , et al. The challenge of periprosthetic joint infection diagnosis: from current methods to emerging biomarkers. Int J Mol Sci. 2023;24:4320. 36901750 10.3390/ijms24054320 PMC10002145

[29] Wang C , Wang Q , Li R , Qin J , Song L , Zhang Q , et al. LTF, PRTN3, and MNDA in synovial fluid as promising biomarkers for periprosthetic joint infection: identification by quadrupole orbital‐trap mass spectrometry. J Bone Jt Surg. 2019;101:2226–2234. 10.2106/JBJS.18.01483 31644522

[30] Xing J , Li J , Yan Z , Li Y , Liu X , He L , et al. Diagnostic accuracy of calprotectin in periprosthetic joint infection: a diagnostic meta‐analysis. J Orthop Surg. 2022;17:11. 10.1186/s13018-021-02895-4 PMC8739654 34991666

[31] Xu H , Xie J , Zhang S , Wang D , Huang Z , Zhou Z . Potential blood biomarkers for diagnosing periprosthetic joint infection: a single‐center, retrospective study. Antibiotics. 2022;11:505. 35453256 10.3390/antibiotics11040505 PMC9030667

[32] Xu Y , Ma X , Guo H , Tang H , Liu J , Wang C , et al. Diagnostic value of synovial fluid biomarkers for periprosthetic joint infection: a prospective, double‐blind trial. Med Sci Monit. 2023;29:e940842 37814443 10.12659/MSM.940842 PMC10578642

[33] Xu Y , Ma X , Guo H , Tang H , Liu J , Wang C , et al. Diagnostic value of synovial fluid biomarkers for periprosthetic joint infection: a prospective, double‐blind trial. Med Sci Monit. 2023;29:e940842. 37814443 10.12659/MSM.940842 PMC10578642

